# Endoscopic drainage with a metallic stent for obstructive jaundice caused by bile duct metastasis of breast cancer: A case report

**DOI:** 10.1002/ccr3.7543

**Published:** 2023-06-13

**Authors:** Kenta Yoshida, Masaki Yokoyama, Yoshinori Hirao, Yuki Sato, Taro Saito, Yasushi Soma, Hiroki Mizukami, Shisaku Fukuda, Hirotake Sakuraba

**Affiliations:** ^1^ Department of Gastrointestinal Medicine and Internal Medicine Kuroishi General Hospital Kuroishi Japan; ^2^ Department of Gastroenterology and Hematology Hirosaki University Graduate School of Medicine Hirosaki Japan; ^3^ Department of Gastrointestinal Surgery and Surgery Kuroishi General Hospital Kuroishi Japan; ^4^ Department of Breast Surgery Tsugaru General Hospital Goshogawara Japan; ^5^ Department of Pathology Kuroishi General Hospital Kuroishi Japan

**Keywords:** bile duct metastasis, breast cancer, SEMS, stenosis

## Abstract

**Key Clinical Message:**

Bile duct metastasis of breast cancer is rare. It often causes obstructive jaundice which makes the patient interrupt state of treatment. Endoscopic drainage for obstructive jaundice is effective and less invasive treatment option also in this case.

**Abstract:**

A 66‐year‐old breast ductal carcinoma patient developed obstructive jaundice, presenting with epigastric discomfort and dark‐colored urine. Computed tomography and endoscopic retrograde cholangiopancreatography revealed bile duct stenosis. Brushing cytology and tissue biopsy confirmed bile duct metastasis, a self‐expandable metallic stent was placed/replaced endoscopically, and chemotherapy was continued, extending the patient's life.

## Graphical abstract

Endoscopic drainage of bile duct was effectively performed and resulted in long‐term survival.

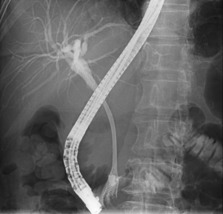

## 1 INTRODUCTION

Obstructive jaundice caused by malignant tumor is commonly encountered in daily practice in the gastrointestinal field. In the cases of cancer metastasis, colon and hepatocellular carcinoma are often the primary lesions, whereas the case of breast cancer is extremely rare.^1^ Herein, we present our experience of a case of obstructive jaundice caused by bile duct metastasis of breast cancer, in which various endoscopic drainage procedures were performed. We believe that this is a valuable case due to the rarity of this presentation, and have therefore reported it with a discussion of the literature.

## 2 CASE HISTORY

**FIGURE 1:**
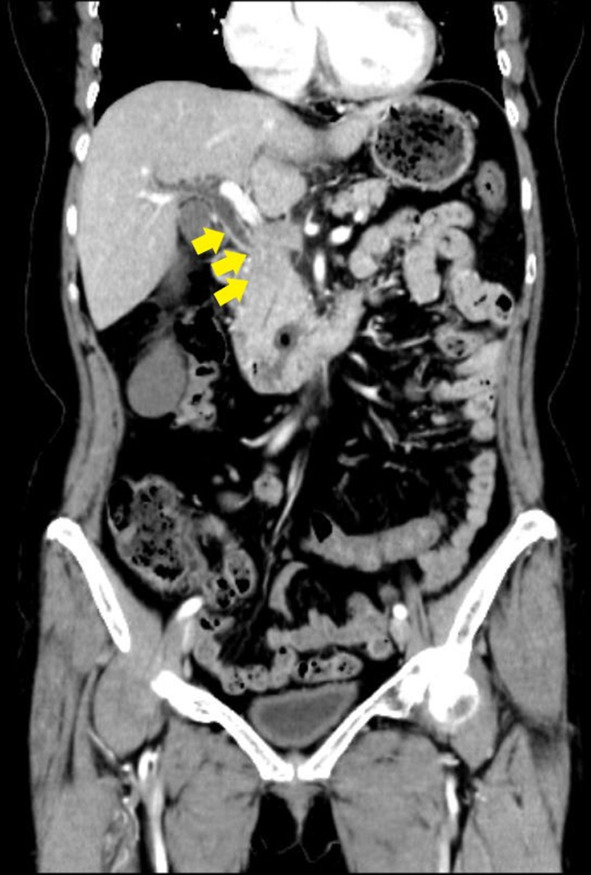
CECT scan of the abdomen showed thickening of the walls of the middle to distal bile ducts (arrows) and dilated intrahepatic bile ducts. No mass was evident around the bile duct wall. CECT, contrast‐enhanced computed tomography.

The patient was a 66‐year‐old woman who had been receiving chemotherapy comprising pertuzumab, trastuzumab, and docetaxel for invasive ductal breast cancer with liver and brain metastases, since 2016. She became aware of epigastric discomfort in October 2019; in early December of the same year, she began passing dark‐colored urine and visited our hospital. Blood tests revealed elevated hepatobiliary enzymes, while contrast‐enhanced computed tomography (CECT) scan of the abdomen showed wall thickening of the middle to distal bile duct and dilated intrahepatic bile ducts. No mass was evident around the bile duct wall (Figure 1). Obstructive jaundice due to malignant lesion in the bile duct was suspected, and the patient was admitted to our hospital for further investigation and treatment.

## 3 DIFFERENTIAL DIAGNOSIS, INVESTIGATIONS, AND TREATMENT

On physical examination, the skin was icteric, and the abdomen was soft, flat, and without any tenderness.

Blood tests revealed elevated hepatobiliary enzymes (total bilirubin, 6.0 mg/dL; aspartate transaminase, 449 IU/L; alanine transaminase, 624 IU/L; alkaline phosphatase, 1560 IU/L; gamma‐glutamyl transferase, 1163 IU/L); inflammatory markers were within normal range (white blood cell, 4600/μL; C‐reactive protein, 0.9 mg/dL); tumor markers were negative (carcinoembryonic antigen, 2.0 ng/mL, carbohydrate antigen 19–9, 2.6 U/mL); and there was no elevation of immunoglobulin G4 (12.1 mg/dL).

**FIGURE 2:**
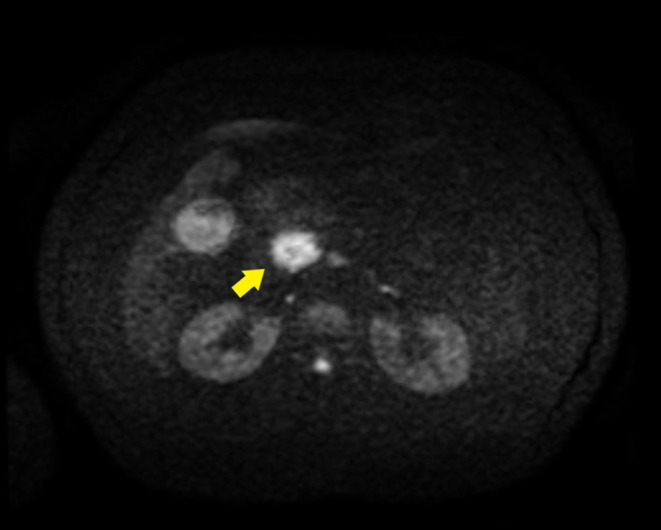
MRI showed a mass‐like lesion around the head of the pancreas that was hyperintense on diffusion‐weighted imaging(arrow). MRI, magnetic resonance imaging.

Magnetic resonance imaging (MRI) performed on the 3rd day of admission revealed a mass‐like lesion around the head of the pancreas that was hyperintense on diffusion‐weighted imaging (Figure 2).

**FIGURE 3:**
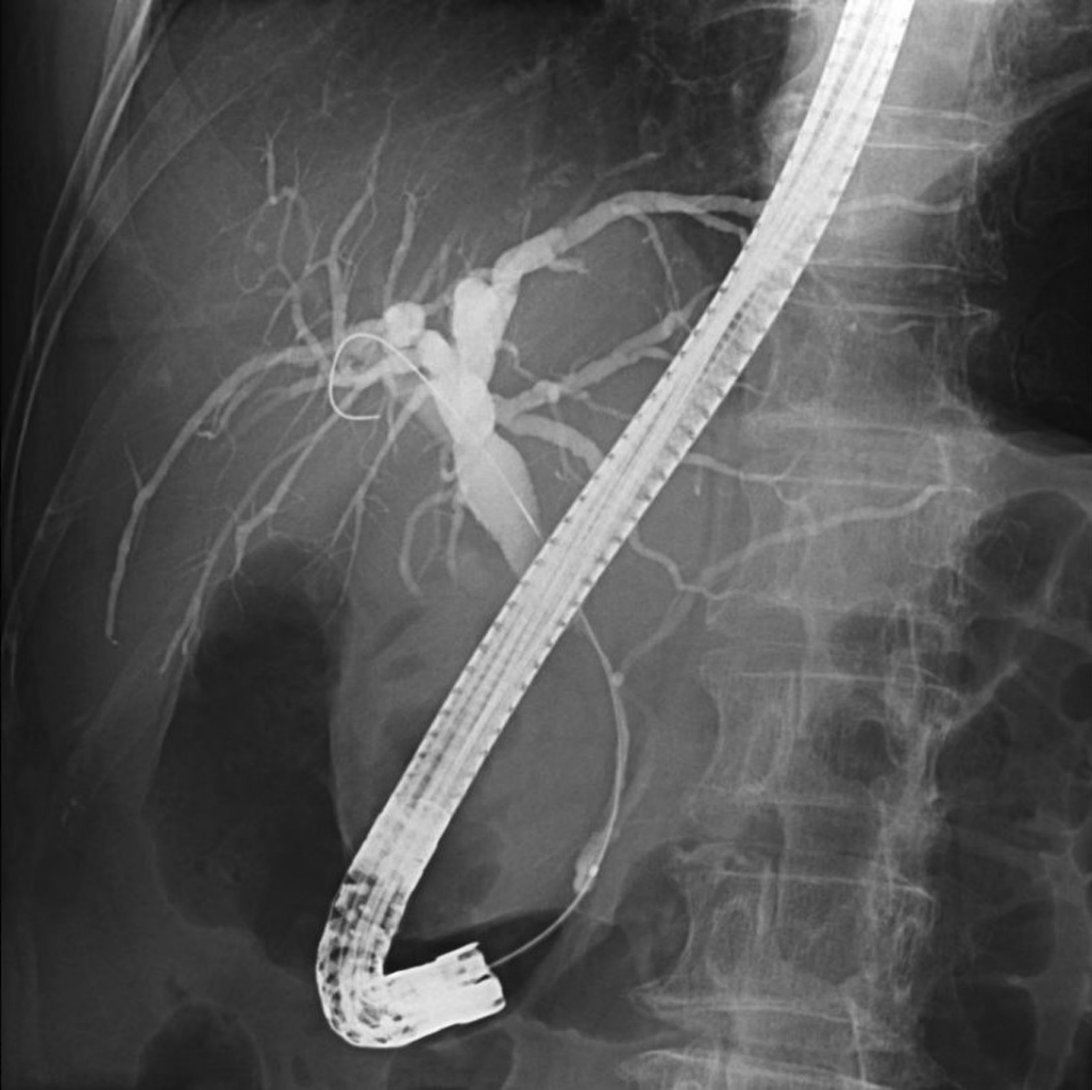
ERCP showed severe stenosis in a relatively long area of the middle to distal bile ducts. ERCP, endoscopic retrograde cholangiopancreatography.

**FIGURE 4:**
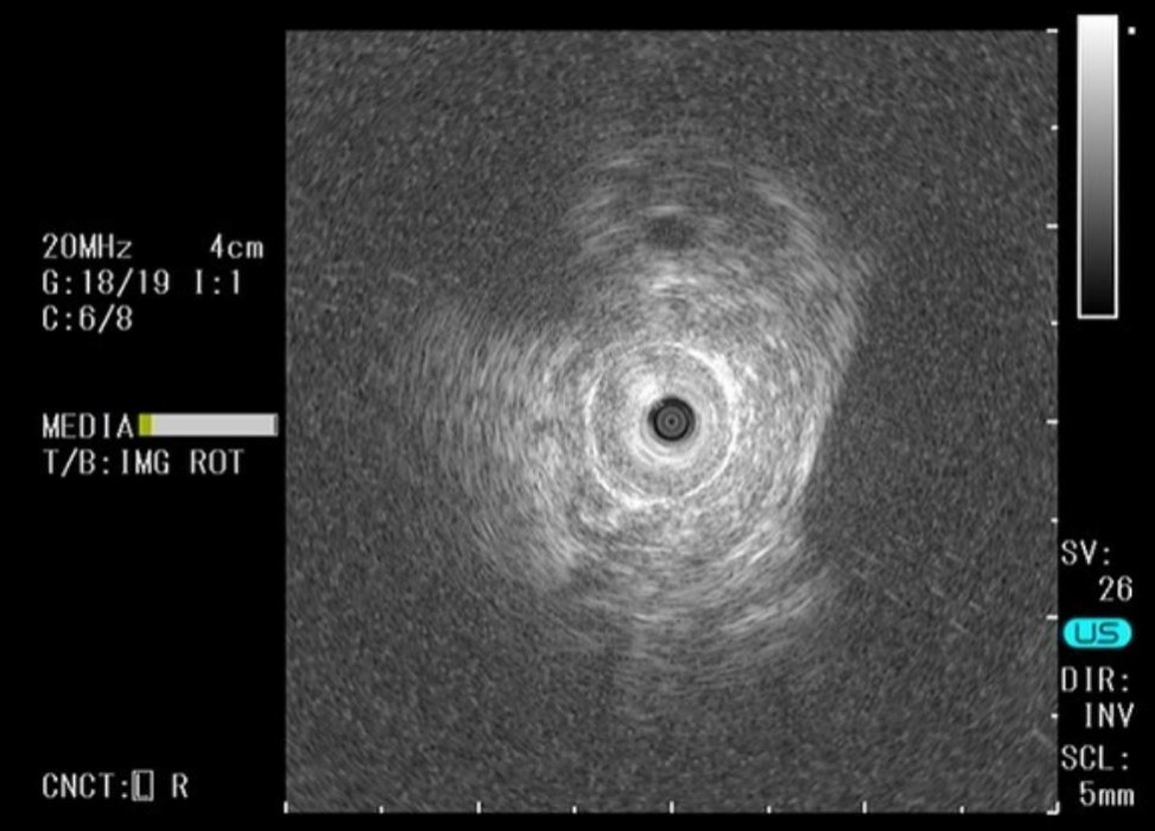
Intraductal ultrasonography showed a homogeneous circumferential thickening (iso‐to hyperechoic) of the bile duct wall at the site of the stenosis was observed.

**FIGURE 5:**
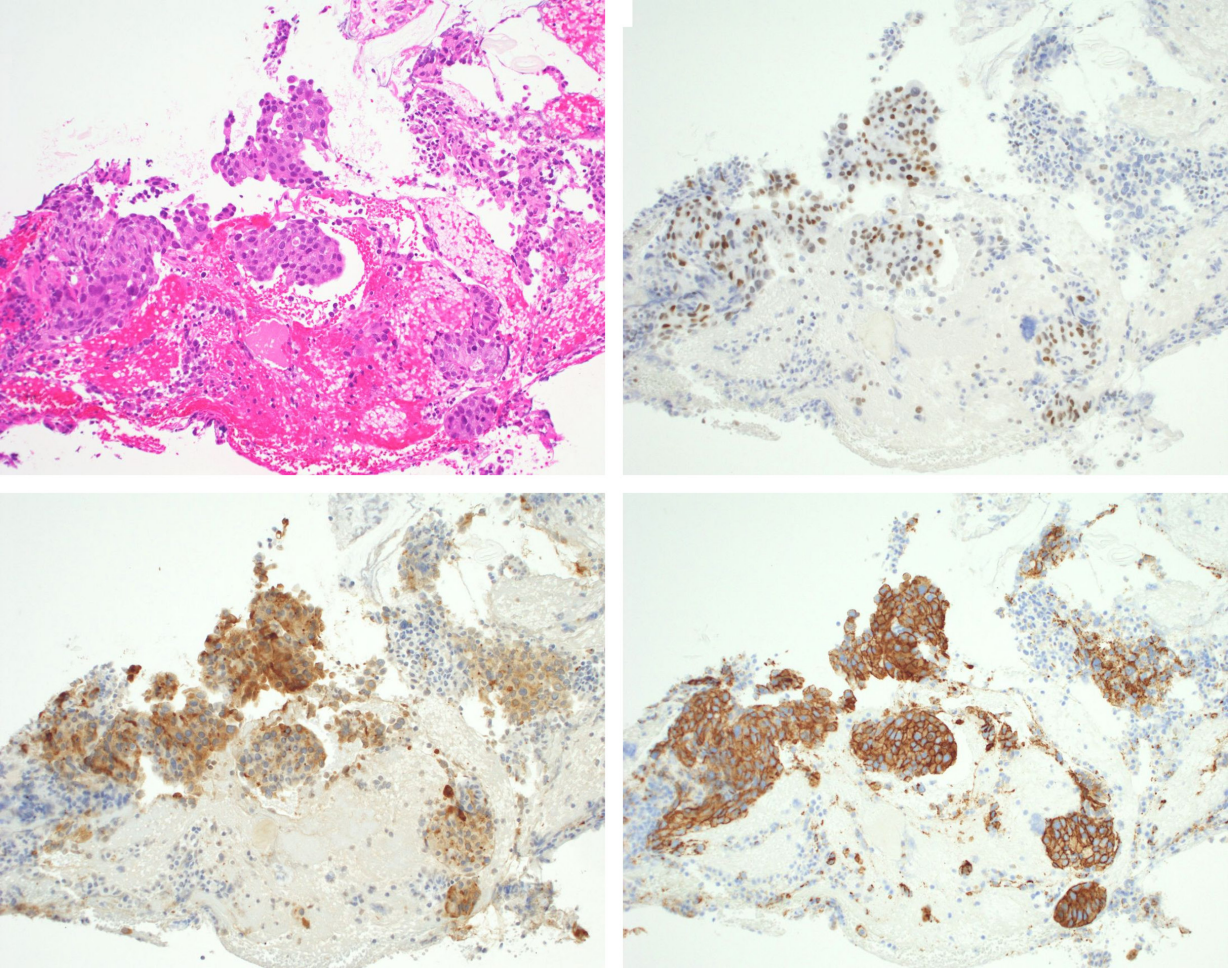
Findings on histopathological examination of the biopsy specimen were suggestive of adenocarcinoma (5‐1), and immunostaining of the same specimen showed GATA3 (5‐2), HER2 (5‐3), and mammaglobin (5‐4) positivity.

On the 5th day of admission, endoscopic retrograde cholangiopancreatography (ERCP) was performed. Cholangiography revealed severe stenosis in an area of the middle to distal bile duct (Figure 3). Endoscopic sphincterotomy was performed, and intraductal ultrasonography revealed homogeneous circumferential thickening (iso‐hyperechoic) of the bile duct wall at the site of stenosis (Figure 4). Brushing cytology and tissue biopsy of the stenotic area were performed, and a plastic stent (straight type, 7 Fr × 90 mm) was placed for drainage. Histopathological findings of the biopsy specimen were suggestive of adenocarcinoma, and immunostaining of the same specimen revealed CK7, GATA3, HER2, and mammaglobin positivity, with CDK2, CK20, PAX8, TTF1, and CA125 negativity, leading to the diagnosis of bile duct metastasis of breast cancer (Figure 5).

## 4 OUTCOME AND FOLLOW‐UP

ERCP‐related complications were not observed, and the patient was discharged on the 9th day of admission, with good progress.

**FIGURE 6:**
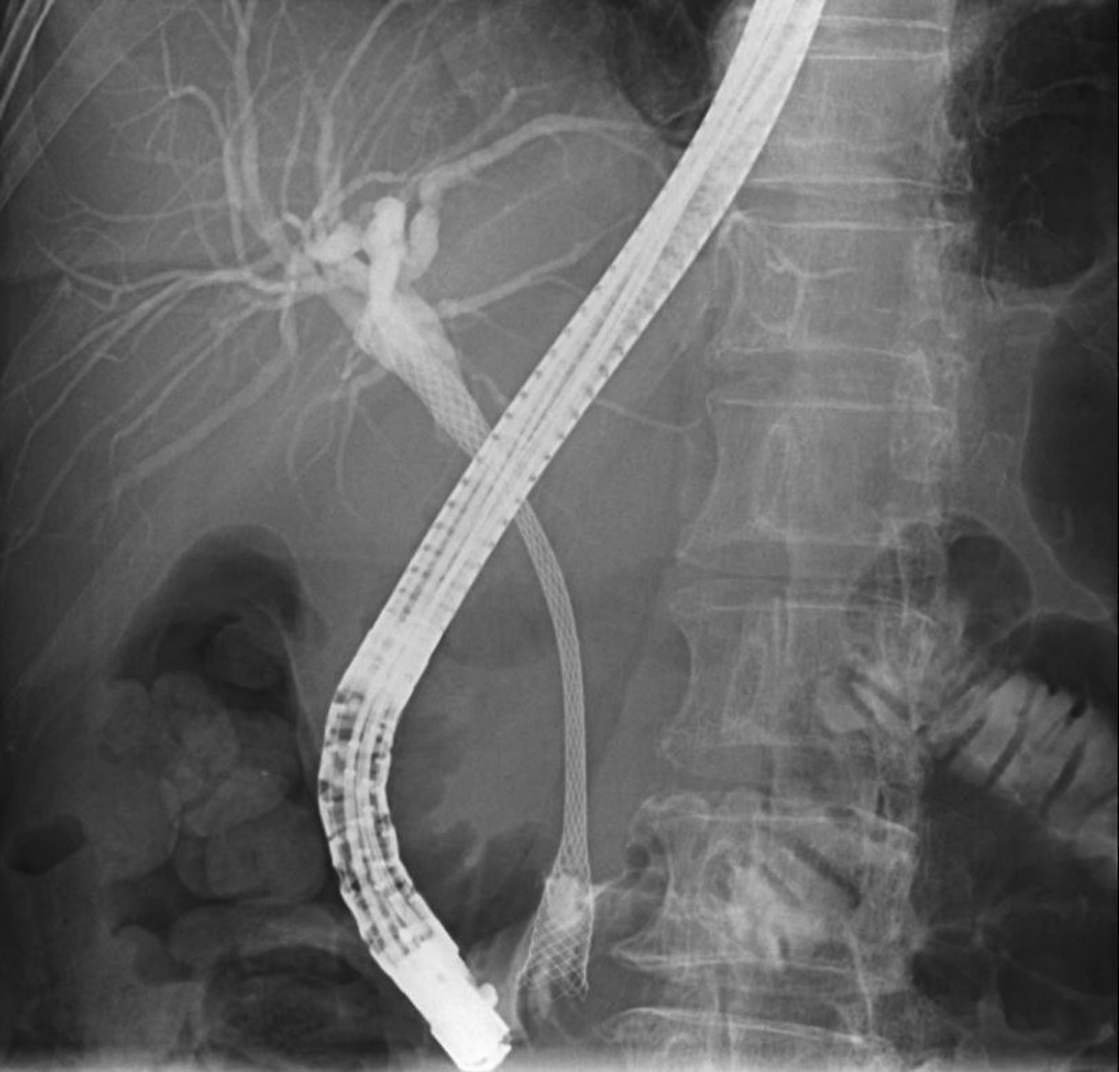
Plastic stent in the bile duct was replaced with a SEMS. SEMS, self‐expandable metallic stent.

In January 2020, the plastic stent in the bile duct was replaced with a self‐expandable metallic stent (SEMS) (WallFlex™ Biliary RX UNCOVERED 10 × 80 mm) (Figure 6). Cholecystitis developed as a complication, which was resolved with percutaneous transhepatic gallbladder aspiration and antibacterial therapy.

**FIGURE 7:**
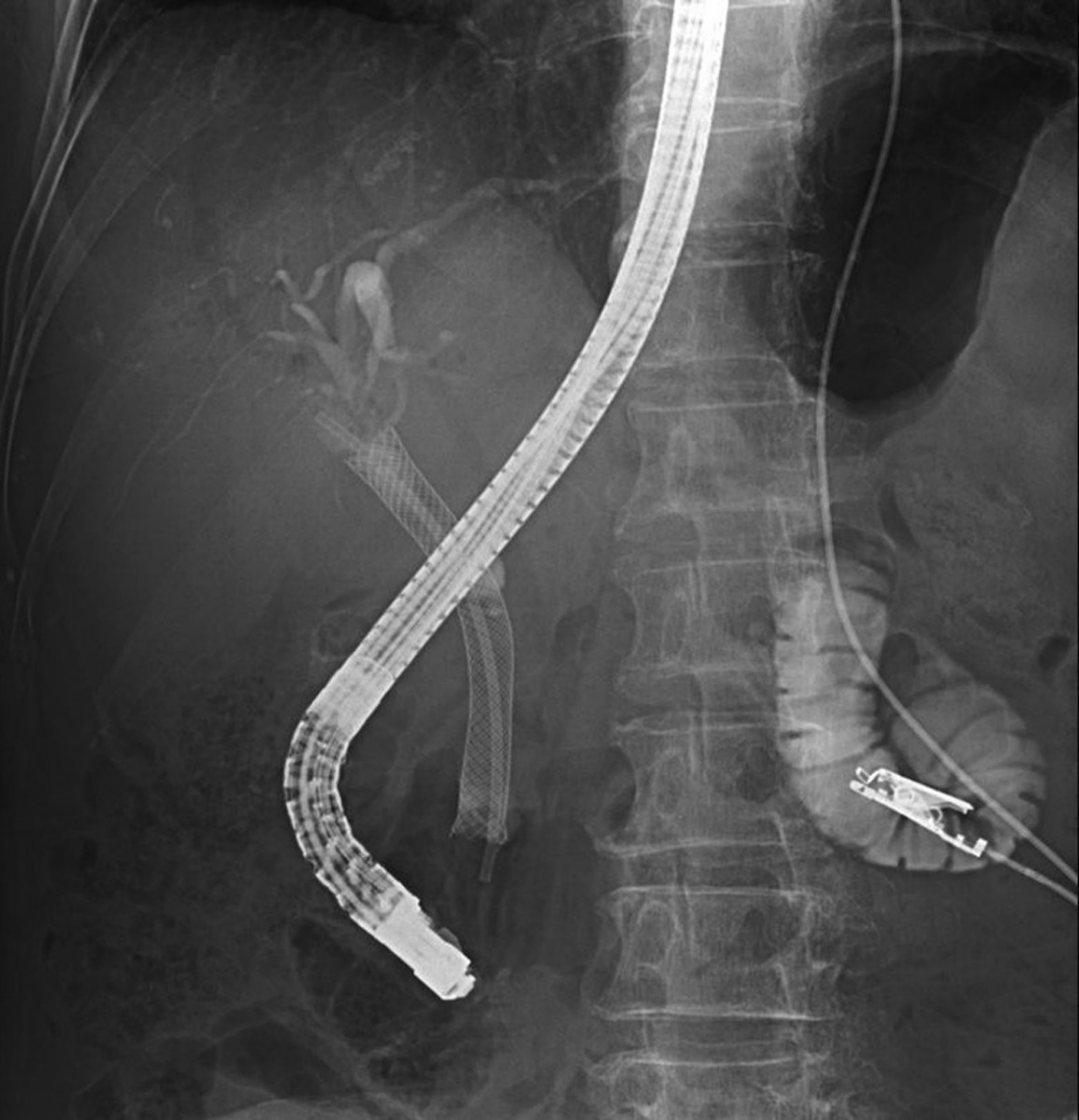
Plastic stent was placed into the SEMS because tumor invasion was suspected. SEMS, self‐expandable metallic stent.

After this treatment, the patient continued chemotherapy as an outpatient but was readmitted in July 2020 due to worsening jaundice. A CECT scan of the abdomen showed soft tissue shadows inside the SEMS, suggesting the possibility of tumor invasion into the SEMS. ERCP was performed, and a plastic stent was therefore placed into the SEMS (Figure 7). Jaundice improved, and the patient was discharged with no complications.

In August of the same year, the patient was readmitted to the hospital for worsening jaundice. ERCP was performed, and the plastic stent, which was suspected to be obstructed, was removed. After the stenosis was dilated with an 8.5 Fr dilation catheter, an alternative plastic stent was placed into the SEMS.

**FIGURE 8:**
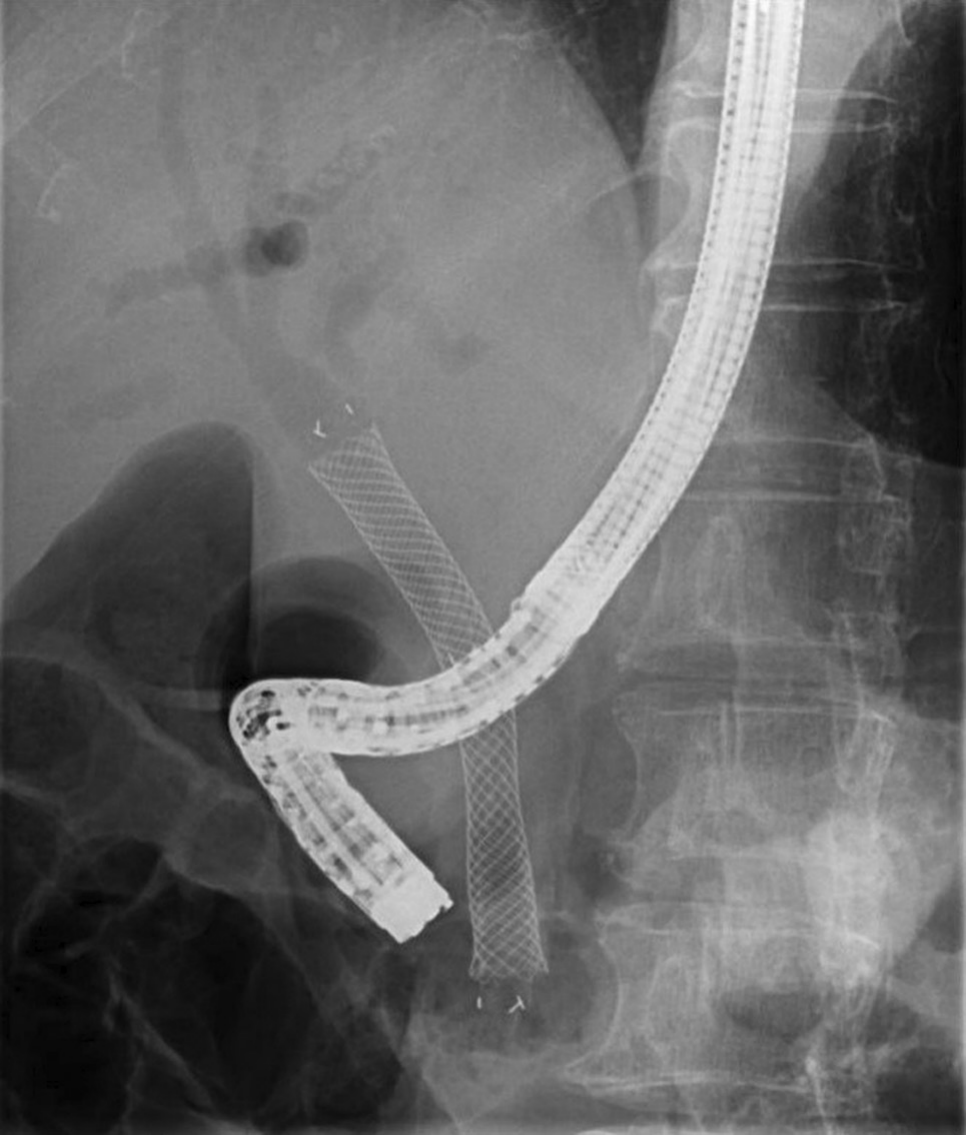
Additional SEMS was placed in the form of a stent‐in‐stent because of severe stenosis in the SEMS due to tumor invasion. SEMS, self‐expandable metallic stent.

In September of the same year, the jaundice worsened again, and the patient was readmitted for further treatment. ERCP was performed, and the plastic stent placed in the SEMS was removed. Severe stenosis was observed in the SEMS due to tumor invasion, and an additional SEMS (HANAROSTNT® Benefit Biliary covered 8 mm × 80 mm) was placed in the stent‐in‐stent configuration (Figure 8).

Thereafter, outpatient chemotherapy was continued. Stent occlusion did not re‐occur, and jaundice did not worsen. However, in December of the same year, her general condition deteriorated due to multiple liver metastases of breast cancer. The patient received palliative treatment and died 358 days after the initial hospitalization.

## 5 DISCUSSION

Breast cancer is the cancer with the highest incident rate in women according to the Global Cancer Statistics in 2020.^2^ Breast cancer often metastasizes to other organs, particularly the lungs, bones, liver, and brain, and, very rarely, the bile duct. Thus far, 16 cases of bile duct metastasis of breast cancer have been reported in detail. These case reports were obtained through a search of PubMed for articles published from 1946 to 2021, using the search terms “breast cancer” and “biliary metastasis.” In addition to these, Japanese case reports were obtained through a search of the Japana Centra Revuo Medicina Web for articles published from 1977 to 2021, using the Japanese equivalents of the search terms mentioned above.^1^, ^3^, ^4^, ^5^, ^6^, ^7^, ^8^, ^9^, ^10^, ^11^, ^12^, ^13^, ^14^ The details of these case reports are presented in Table 1. The average age of the patients was 56 years (42–70 years). Most cases were examined for subjective symptoms, such as jaundice (11/16), abdominal pain (3/16), and pruritus (3/16), and only one case was asymptomatic. The mean time to metastasis to the bile duct was 7.4 years (2–21 years), and most of the cases further showed metastasis to other organs such as the gallbladder, liver, and bones. Many of the reported cases required multiple examinations for diagnosis. In reports before the 1990s, most cases were diagnosed by histology of resected specimens after surgery; however, recently, some cases were diagnosed by ERCP.

**TABLE 1:** Cases of bile duct metastasis of breast cancer.

Case	Reporter	Year	Age	Symptom	Other metastatic organ	Number of years to bile duct metastasis	Examination						Diagnosis	Treatment for jaundice	Prognosis (time since treatment)
							ERCP	US	CT	MRI	PET	PTC			
1	Our case	2021	66	Jaundice, urine concentration	Liver, brain, LN	3	+		+	+			Biopsy, cytology	Endoscopy (PS, SEMS)	Dead(1 year)
2	Jie	2021	55	Abdominal pain	Skin, bone	3	+	+		+			Biopsy	Endoscopy (PS)	Dead
3	Hatano	2020	55	Abdominal pain, fever	Bone, LN	9	+		+	+			Cytology	Endoscopy (PS)	Alive (1.5 years)
4	Shima	2020	67	Jaundice	Bone, LN	19			+	+	+		Surgery	Surgery	Alive (1 year)
5	Justin	2015	70	Jaundice, nausea, body weight loss	Pancreas, subcutaneous, muscle, tongue, LN	21		+	+		+		Fine‐needle aspiration	Endoscopy (PS)	Unknown
6	Ivan	2015	49	Pruritus	Pancreas, LN	6	+	+			+		Cytology	Endoscopy (PS, SEMS)	Unknown
7	Coletta	2014	56	Jaundice	LN	13	+	+	+	+			Cytology, surgery	Surgery	Alive
8	Sasaki	2005	56	None	LN	5	+		+	+			Surgery	Surgery	Alive (2 years)
9	Titus	1997	50	Pruritus, diarrhea	Vater papilla	4	+	+	+				Surgery	Surgery	Alive
10	Engel	1997	48	Jaundice, pruritus, urine concentration	Liver, lung, bone	4		+				+	Surgery	Surgery	Unknown
11	Papo	1996	58	Jaundice, body weight loss	None	10	+	+	+				Surgery	Surgery	Dead (1 year)
12	Pappo	1991	52	Jaundice, abdominal pain	Gallblader, pancreas, subcutaneous	2		+	+				Surgery	Surgery	Alive (1 year)
13	Franco	1987	54	Jaundice	LN	6		+				+	Surgery	Surgery	Dead (4 years)
14	Franco	1987	42	Jaundice	Bone	8		+					Surgery	Surgery	Alive (2.5 years)
15	Rabin	1979	69	Jaundice, appatite loss	Gallblader, liver, peritoneum	2						+	Surgery	None	Dead (3 days)
16	Rabin	1979	46	Jaundice	Liver	4		+				+	Image only	None	Dead (4 weeks)

Abbreviations: PS, plastic stent; PTC, percutaneous transhepatic cholangiography; SEMS, self‐expandable metallic stent.

Regarding treatment for objective jaundice by bile duct stenosis, only five cases were treated by endoscopic drainage (Table 2).^1^, ^3^, ^4^, ^6^ To patients who might respond to other modes of therapy enabling longer survival, surgical bypass could be more effective than endoscopic treatment.^12^ But in our case, endoscopic drainage was chosen because metastases were found in multiple organs other than the bile ducts, so long‐term prognosis was not promising, and also because of the invasiveness of surgery. On cholangiography, all the reported cases with available images showed severe diffuse stenosis confined to a relatively long segment of the bile duct. In four of these cases, tissue biopsy or brushing cytology at the stenotic area was performed, all of which led to a definitive diagnosis. Conversely, Coletta et al. reported a case of bile duct metastasis of breast cancer, in which endoscopic cytological examination of the stenotic area was negative; histologic examination of the resected specimen after surgery revealed tumor cells on the outer side of the bile duct, but not on the bile duct endothelium.^7^ In such cases, it is difficult to make a definitive diagnosis based on endoscopic bile duct biopsy or brushing cytology alone; therefore, a comprehensive approach based on clinical history and findings on other imaging modalities are required. Our case is the first to describe the duration of stent patency, and in which stent stenosis due to tumor invasion was observed repeatedly within a short period. There was a previous case report of gastric metastasis of breast cancer, in which an SEMS was placed for pyloric stenosis, and obstruction due to tumor invasion was observed barely 3 months later.^15^ This indicates that in the case of breast cancer metastasis, in‐stent invasion may occur earlier. Furthermore, it may be useful to place SEMS in the early stages after diagnosing biliary metastasis, as it is superior to plastic stent from the viewpoint of stent patency. Regarding complications, cholecystitis occurred in two of reported cases after endoscopic stenting; however, other serious adverse complications were not reported. Prognosis varied from case to case, depending on the degree of progress of breast cancer and metastases.

**TABLE 2:** Obstructive jaundice caused by bile duct metastasis from breast cancer treated with endoscopic drainage.

Case	Reporter	Year	Age	ERCP Image	Definite diagnosis	Stent type	Stent patency period	Accident after stenting	Post treatment	Prognosis
1	Our case	2021	66	Localized diffuse stenosis	Biopsy	Plastic stent → SEMS	7 months	Cholecystitis	Chemotherapy	Dead (1 year after ERCP)
2	Jie	2021	55	Localized diffuse stenosis	Biopsy	Plastic stent	Unknown	–	Chemotherapy	Dead (date unknown)
3	Hatano	2020	55	Localized diffuse stenosis	Cytology	Plastic stent	Unknown	Cholecystitis	Chemotherapy	Alive (1.5 year after ERCP)
4	Justin	2015	70	No image	Puncture suction	Plastic stent	Unknown	–	Unknown	Unknown
5	Ivan	2015	49	Localized diffuse stenosis	Cytology	Plastic stent → SEMS	Unknown	–	Endocrine therapy	Unknown

In summary, we encountered a rare case of bile duct breast cancer metastasis, in which obstructive jaundice was effectively managed by endoscopic drainage. Endoscopic bile duct stenting is less invasive than surgical operation, making it an effective treatment option.

## AUTHOR CONTRIBUTIONS

**Kenta Yoshida:** Conceptualization; data curation; visualization; writing – original draft. **Masaki Yokoyama:** Investigation; resources. **Yoshinori Hirao:** Investigation; resources. **Yuki Sato:** Project administration; supervision. **Taro Saito:** Project administration; supervision. **Yasushi Soma:** Project administration; supervision. **Hiroki Mizukami:** Investigation; resources. **Shinsaku Fukuda:** Supervision. **Hirotake Sakuraba:** Supervision.

## FUNDING INFORMATION

The author receives no research funding.

## CONFLICT OF INTEREST STATEMENT

The authors declared no conflict of interest.

## ETHICS STATEMENT

This report was conducted according to the principles of the Declaration of Helsinki.

## CONSENT

Written informed consent was obtained from the patient to publish this report in accordance with the journal's patient consent policy.

## Data Availability

Data sharing is not applicable to this article as no new data were created or analyzed in this study.
